# Male mate choice in mosquitofish: personality outweighs body size

**DOI:** 10.1186/s12983-022-00450-3

**Published:** 2022-01-21

**Authors:** Chunlin Li, Xinyu Zhang, Peng Cui, Feng Zhang, Baowei Zhang

**Affiliations:** 1 School of Resources and Environmental Engineering, Anhui University, No.111, Jiulong Road, Hefei, 230601 China; 2 Anhui Province Key Laboratory of Wetland Ecosystem Protection and Restoration, Anhui University, No.111, Jiulong Road, Hefei, 230601 China; 3 Nanjing Institute of Environmental Sciences, Ministry of Ecology and Environment, No.8, Jiangwangmiao Road, Nanjing, 210042 China; 4 School of Life Sciences, Anhui University, No.111, Jiulong Road, Hefei, 230601 China

**Keywords:** Activity, Animal personality, Male mate choice, Mosquitofish, Shyness

## Abstract

**Background:**

Despite its important implications in behavioural and evolutionary ecology, male mate choice has been poorly studied, and the relative contribution of personality and morphological traits remains largely unknown. We used standard two-choice mating trials to explore whether two personality traits (i.e., shyness and activity) and/or body size of both sexes affect mate choice in male mosquitofish *Gambusia affinis.* In the first set of trials involving 40 males, we tested whether males would prefer larger females and whether the preference would be affected by males’ body length and personality traits, and females’ activity level. In the second set of trials (using another 40 males), we tested whether males would prefer more active females and whether the preference would be affected by males’ body length and personality traits.

**Results:**

Both shyness and activity in males were significantly repeatable and constituted a behavioural syndrome. No overall directional preference for large (or small) females with the same activity levels was detected because larger males preferred larger females and smaller males chose smaller females. Males’ strength of preference for larger females was also positively correlated with the activity level of larger females but negatively with the activity level of smaller females. Males spent more time associating with active females regardless of their body lengths, indicating males’ selection was more influenced by female activity level than body size. Males’ preference for inactive females was enhanced when females became active. There was no convincing evidence for the effect of males’ personality traits or body length on their preferences for females’ activity level.

**Conclusions:**

Our study supports the importance of body size in male mate choice but highlights that personality traits may outweigh body size preferences when males choose mating partners.

**Supplementary Information:**

The online version contains supplementary material available at 10.1186/s12983-022-00450-3.

## Background

As females generally invest more than males in their offspring, they are usually considered to be highly selective when choosing their mates. Therefore, studies on mate choice have mostly focused on female choice of male mates and its implications for the evolution of male ornaments [1, 2]. However, increasing evidence suggests that male mate choice is also widespread in many taxa, and possible explanations include paternal investment by males [3, 4], males’ greater mating effort [5], differences in female quality [6], female-biased operational sex ratio [7], and sperm production limits [8]. Studies have highlighted that, like female mate choice, male mate choice has ecological and evolutionary significance, such as increasing population adaptation to new environments [9, 10] and accelerating sympatric speciation through reproductive isolation [11, 12].

Compared to female mate choice, less is known about the way in which males choose their mates, or the female traits that are targeted by male choice although there has been an increasing interest in male mate choice in the last few decades [9, 13]. From an evolutionary point of view, mate choice should be based on traits that maximize the fitness of the individual and these selected traits would be driven evolutionarily by mate choice [14]. Previous studies on female mate choice have investigated a variety of male traits that may influence female mate choice, such as ornaments, social dominance, body size, cognition, and chemical odours [15–17]. Males may also use these traits when selecting mates; however, this field is comparatively less well studied [9].

Body size is a widely studied trait that is used in mate selection by both females and males in many species, with larger mating partners being preferred [18–20]. Larger size often confers advantages to males in resource defending, intrasexual competition, and social dominance. Moreover, there may be genetic and ecological advantages for females to mate with larger males [18, 21, 22]. Female body size is often positively correlated with their fecundity [17, 23], and highly selective males can increase reproductive success by preferentially mating with larger females. This has been empirically found in some live-bearing fish species [17, 19, 24]. In addition, both males and females in some species exhibit assortative mating by body size [22, 25].

During the last few decades, evidence of animal personality has been widely found throughout the animal kingdom [26, 27]. Researchers often measured one or more of five personality traits proposed by Réale et al. [28], i.e., shyness-boldness, exploration, activity, aggressiveness and sociability. Some studies have suggested that personality may be a behavioural criterion influencing mate choice decisions in females and males [29–31]. Personality traits can be linked with fitness and thus are potential targets of natural and sexual selection [26]. For example, survival is positively correlated with activity level in the wild brown trout *Salmo trutta* [32]. Activity level has also been found to be positively correlated with rates of resource intake, and thus positively correlated with growth or reproduction [33]. To maximize reproductive success, males are predicted to choose females with personality traits that can result in higher reproductive success, for example, a higher activity level [34]. Meticulousness regarding personality may also depend on the selectors’ own personality traits [35]. In addition, personality may covary with body size [36, 37], and thus these two phenotypic traits may interact to influence mate choice in both sexes. For example, more active females in *Poecilia mexicana* exhibit stronger mating preferences for larger males [31]. Exploring the role of personality in mate choice can aid our understanding of how sexual selection contributes to maintaining personality and the relative importance of personality and morphological traits (e.g., body size) in mate choice. However, to date this field is still understudied, with limited research on the mating preferences of females, and even less data on those of males [29, 30, 38].

In this study, we used mosquitofish *Gambusia affinis* to evaluate whether personality and body size affect male mate choice. We first characterized personality traits in male mosquitofish, including shyness and activity. These males were subsequently tested in dichotomous mate choice experiments in which females with various combinations of activity levels and body sizes were used as stimuli. As observed in many other poeciliid fish species [39–42], we expected male fish to prefer larger females with higher fecundity. Because females often resist males attempting to mate due to sexual harassment [43], we further expected that the preference for larger females would be stronger in larger males that can more easily overcome the stronger resistance of larger females. Activity level is a positive proxy of female quality which is related to females’ reproduction success [32, 33], and thus we expected that males would preferentially select females with higher activity levels. Because more proactive (bolder and more active) males have higher competition ability and can better overcome the resistance of active females [43], we further expected that more proactive males would have stronger preferences for more active females.

## Methods

### Study animals

Mosquitofish is a poeciliid fish species native to North America, and it has been intentionally introduced in many countries with the aim of controlling mosquitos [43]. Poeciliid fish are characterized by promiscuity, internal fertilization, ovoviviparity, and sexual dimorphism, with males being smaller than females [44]. At sexual maturity, female mosquitofish possess two gravid spots on the posterior of their abdomens, while males have a gonopodium modified from the anal fin. The mating system in this species is non-resource-based and promiscuous. Males do not court, but instead, sneakily approach females from behind and attempt coerced copulations [43]. After internal fertilization, the fertilized ova hatch within the female ovary in 22–25 days [43]. The brood size depends on mother’s body size, with larger ones giving birth to more newborns, which are approximately 6–8 mm in length [43]. The time it takes fry to reach sexual maturity varies from one month to several months, depending on the water temperature. Standard body length at sexual maturity is usually larger than 15 mm in males and 17 mm in females [43].

A total of 1500 newborns generated from 150 wild-caught females were uniformly reared in 30 net tanks (80 × 80 × 80 cm, mesh size: 0.177 mm) in an outdoor artificial pond on the campus of Anhui University from May to July 2018 [45]. There was an additional net tank containing a group of approximately 200 newborns from which males were excluded as soon as they could be sexually identified. This generated virgin females used as stimuli in the mate choice experiments. The fish were fed brine shrimp nauplii until two weeks old and thereafter fine-grained commercial food (TIDDLER, Weifang YEE Pet Products Co., Ltd., China; 42% crude protein, 5% crude fat, 5% crude fibre, and 11% ash). The water temperature during the rearing period ranged from 20 to 32 °C, and the pH ranged from 7.4 to 7.6. Apart from the additional food, the rearing conditions were the same as those of their conspecifics already living outside the tanks in the pond for several years, avoiding behavioural abnormalities that might arise in laboratory conditions [46].

In July, 80 sexually mature males (standard body length > 15 mm), indicated by a clear apical hook at the gonopodium tip [43], were randomly net caught from the rearing tanks and were randomly divided into two groups of 40 individuals. One group was allocated to Experiment 1 where the effect of female body size on mate selection was investigated, and the other group to Experiment 2 (Table 1) where the effect of female activity level on mate selection was explored. To avoid any non-experimental stimuli, the males were individually kept in black, opaque, cylindrical tanks (height: 9 cm; diameter: 15 cm; hereafter, holding tank) with a black, opaque, cylindrical refuge chamber (height: 5 cm; diameter: 7 cm) placed in the centre. The holding tanks were filled with oxygenated tap water, and the fish were acclimated to the chamber for more than 24 h before the experiments. Then each group of 40 males were randomly divided into 10 subgroups of four that were tested separately with different healthy, active, virgin females as stimuli. In Experiment 1 each subgroup of males was exposed to a pair of mature females with different body lengths (22 mm vs. 18 mm). In Experiment 2 each subgroup was exposed to a pair of mature females with the same body length (22 mm), but different activity levels. The intensity and size of gravid spots are linked with developmental stages and clutch size of female live-bearing fish and thus may influence mate choice of males [47, 48]. Consequently, we paired females with similar sizes and intensities of gravid spots. During experiments, because fish were always handled in water and were always given enough time to acclimate, no stress responses were observed in the stimulus females.

**Table 1:** Overview of experiments on male mate choice in mosquitofish *Gambusia affinis*

Experiment	Hypotheses	Trial	Stimulus females
1	1. Male fish prefer the larger female and the preference is stronger in larger males 2. Males preferentially select the active female regardless of whether it is larger or smaller, selecting female’s activity over its body size	1–1	22-mm active versus 18-mm active
		1–2	22-mm active versus 18-mm inactive
		1–3	22-mm inactive versus 18-mm active
		1–4	22-mm inactive versus 18-mm inactive
2	Males preferentially select the active female and the preference is stronger in more active males	2–1	22-mm inactive versus 22-mm active
		2–2	22-mm active versus 22-mm active

Eighty male individuals were divided into two groups of 40. Each of the 40 males underwent 4 two-choice mating trails in Experiment 1, where the effect of female body size on male mate choice was investigated and each of the other 40 males underwent 2 trials in Experiment 2 where the effect of female activity level on male mate choice was investigated

To mimic two levels of female activity for males to choose from, females were restricted (inactive female) or not (active female) by a transparent plastic cylinder (6.5 cm diameter × 8 cm high) as required during the experiments. After a period of acclimation, the inactive females adapted to the trap and lowered their activity levels with no obvious abnormal behaviours. Instead of measuring females’ actual activity levels that may vary significantly and not as expected during experiments, this experimentally manipulated difference in activity levels between stimulus females could generate more robust results. The cylinders used to trap the females were transparent and were separated by plexiglass. Therefore, males could not perceive that the active females were constrained.

### General experimental procedure

To test the effect of the personality of male mosquitofish on their mate choice, open arena assays were used, to measure shyness and activity twice for each male in two successive days (see the subsection of personality measurements for details). Subsequently, mate choice experiments were carried out for each of the two groups of 40 males, respectively (see the subsection of mate choice experiments for details). The first mate choice experiment (Experiment 1, Table 1) tested males’ preferences regarding body size (22 mm vs. 18 mm) of mature females and whether male preference was influenced by female activity levels (active vs. inactive) and male personality traits. The second experiment (Experiment 2, Table 1) tested males’ preference regarding females with different activity levels (active vs. inactive), but the same body size and whether their preferences changed when the inactive female increased her activity level.

**Fig. 1:**
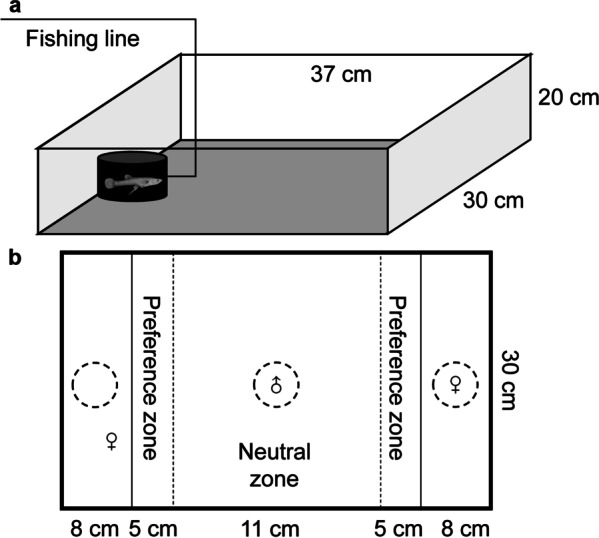
Experimental apparatus for measuring **a** shyness and activity, and **b** mate preference of male mosquitofish *Gambusia affinis*

The experiments were carried out in a white opaque plastic tank (37 cm long × 30 cm wide × 20 cm high, Fig. 1) in the same laboratory under enough light and constant temperature (26 °C). A camera (Sony HDR-CX510, 55× extended zoom, Sony Corporation, Tokyo, Japan) was fixed above the tank to record the behaviours of the subjects throughout. To avoid potential disturbances, the experimenters were shielded from the experimental apparatus by a 1.5-m high opaque curtain during each trial. To minimize observer bias, blinded methods were used when behavioural data were recorded and/or analysed. At the end of all experiments, each male was gently placed against the transparent wall of a glass tank to measure its standard body length (accurate to 0.1 mm), and all the fish were released to the rearing pond.

### Personality measurements

In the assays for shyness and activity, a black, opaque, cylindrical refuge chamber (the same refuge placed in the holding tank, hereafter, starting refuge) was fixed at the far end of the experimental tank (Fig. 1a). A sliding trapdoor (3 × 3 cm) on the side of the starting refuge facing the arena was connected to a piece of fishing line that allowed experimenters to remotely open the refuge to allow fish to emerge from the chamber and move toward the arena without disturbance. The tank was filled with oxygenated tap water to a depth of 3 cm, and the water was exchanged after each subject was tested. Males were not fed for more than 12 h before the personality assays.

At the beginning of the assay, a male was gently transferred from the holding tank to the closed starting refuge in the arena. The subject was allowed to acclimate for 5 min, and then the trapdoor of the refuge was remotely opened and was not closed until the end of the trial. Following previous studies [49–51], shyness was measured as the time taken by the subject to swim out of the refuge, that is, for its whole body to cross the trapdoor. All the subjects emerged from the refuge within 6 min. After the male left the refuge, it was video-tracked continuously for 10 min to record its movement trajectory. At the end of the trial, the subject was immediately transferred to its holding tank. A total of 600 image stacks were extracted from the 10-min movement videos (one frame per second); Image J (http://rsbweb.nih.gov/ij/) was used to delineate each subject’s movement pathway, of which the total length was used to quantify its activity level.

### Mate choice experiments

To carry out the mate choice trials in a dichotomous chamber, the white opaque plastic tank was divided into three compartments separated by plexiglass, only allowing visual contact between fish in different compartments. At the centre of each compartment, there was a transparent plastic cylinder (6.5 cm diameter × 8 cm high) connected to a piece of fishing line, by which the experimenters could remotely pull up the cylinder to allow the trapped fish to swim freely. Two dark lines were drawn on the bottom of the tank to demarcate a neutral zone (11 cm wide) and two preference zones (5 cm wide; Fig. 1b). The tank was filled with 7-cm-depth oxygenated tap water, which was changed after each trial. One hour before the mate choice experiments, males were fed to avoid the effect of hunger.

*Experiment 1* In this experiment, four successive trials (Table 1) were designed to study whether males’ personality and females’ activity level influence male preference for female body size. Firstly, two females (22-mm active female; 18-mm active female) were separately placed outside the cylinders in the end compartments of our experimental setting, and a male was trapped inside the cylinder in the central compartment. After a 10-min acclimatization and observation period, the male was remotely released to allow it to choose between the two females, and its movement behaviour was video-tracked for 12 min (Trial 1–1, Table 1). Secondly, the male and the smaller female were trapped inside their cylinders, while the larger female was outside the cylinder. After 10 min for acclimatization, the male was released to swim freely and was video-tracked for 12 min (Trial 1–2, Table 1). Thirdly, the male and the 22-mm female were trapped, while the 18-mm female swam freely. After 10 min, the male was released and video-tracked for 12 min (Trial 1–3, Table 1). Fourthly, the male and the two females were all trapped initially, and after 10 min, the male was released and video-tracked for 12 min (Trial 1–4, Table 1). To provide the same experimental conditions to all males, they underwent the same order of the above treatments, which may have mask effect on the treatments (i.e., the order effect). However, the randomized orders of the focal males and stimulus females, and the 10-min acclimatization between treatments may reduce the possible order effect.

*Experiment 2* Two trials were used to explore whether males (the other group of 40 individuals) choose mating partners according to males’ personality traits and females’ activity level. Firstly, two stimulus females of the same body size (22 mm) were separately placed in the end compartments, with the inactive female restricted inside its cylinder while the active female freely swimming outside. A male was restricted inside the cylinder in the central compartment for 10 min to acclimate and observe the activities of both females. Then, the experimenter allowed the male to swim around freely and video-tracked its movement for 12 min (Trial 2–1, Table 1). Secondly, the male was restricted inside the cylinder again, and the inactive female was released to swim freely. After 10 min for acclimatization, the male was released and video-tracked for 12 min (Trial 2–2, Table 1). To avoid potential side-biases, the locations of the two females were exchanged between trials.

### Statistical analyses

Following Dingemanse and Dochtermann [52], a bivariate linear mixed-effects model was fitted using the R package *MCMCglmm* (*v.* 2.29) for the males used in Experiment 1 and 2, respectively, to measure the repeatability of each behaviour (i.e. personality) and the among-individual correlation between the two behaviours (i.e. behavioural syndrome). The two log_e_-transformed behavioural traits were concurrently included as the response variables in each model with individual ID as a random effect. The bivariate models were run for 220,000 iterations after 20,000 burn-in iterations and were thinned by 25 iterations. Model convergence was confirmed by the Gelman–Rubin diagnostic test (function *gelman.diag*).

Male mate preference for a particular female was determined by noting the association time, that is, the time a male spent within the preference zone near each stimulus female. Association time is widely used to measure mating preferences in female and male live-bearing fish [17, 53–57]. To further avoid disturbances from experimental operations, the middle 10 min of each 12-min free-choice period was used for the analyses. Time 1 (T1) was defined as the association time of the male with the large (or active) female and T2 with the small (or inactive) female. Male’s strength of preference (SOP) for large versus small (in Experiment 1, Table 1) or active versus inactive females (in Trial 2–1, Table 1) was calculated as: SOP = (T1 − T2) / (T1 + T2). The SOPs ranged from − 1 to 1, with larger values indicating a stronger preference for large (or active) females. The SOPs were tested using the Shapiro–Wilk test and were found to be normally distributed (*p* = 0.179 in Experiment 1 and *p* = 0.257 in Trial 2–1).

The mean values of shyness and activity measured for each male in the two personality trials were used in the following analyses. A *t*-test was used to test the differences in body length, shyness, and activity between the males used in Experiment 1 and 2. The repeated-measures ANOVA in R package *nlme* (*v.* 3.1–148) and post hoc Tukey’s test with a Bonferroni correction were used to test the differences in time spent by males in mate choice assays among the three different zones (i.e. neutral zone and the two preference zones). A paired *t*-test was used to investigate the change in males’ association time with the inactive female in Trial 2–1 after it was released to be active in Trial 2–2.

Generalized linear mixed models with log link functions were developed using R package *lme4* (*v.* 1.1–23) to fit the effects of explanatory factors on male mate preferences (SOPs as the dependent variables) for the larger (Experiment 1) or the active female (Trial 2–1). Body length, shyness and activity of the males and activity levels of the two stimulus females were identified as fixed effects, and the male ID, trial ID and pair ID of stimulus females as the random effects in the model for Experiment 1. The model for Trial 2–1 had the pair ID of stimulus females as the random effect and male’s body length, shyness, and activity as the fixed effects. There were no significant correlations between males’ body length and shyness (Pearson correlations, Experiment 1: *r* = 0.25, *t* = 1.57, *df* = 38, *p* = 0.125; Experiment 2: *r* = 0.07, *t* = 0.45, *df* = 38, *p* = 0.656) or activity (Experiment 1: *r* = -0.16, *t* = -1.02, *df* = 38, *p* = 0.314; Experiment 2: *r* = -0.13, *t* = -0.80, *df* = 38, *p* = 0.431). All analyses were carried out using R 4.0.2 [58], and data are displayed as mean ± standard error.

## Results

### Body length and personality of males

The body lengths of males tested in Experiment 1 (18.2 ± 0.3 mm, range: 15–22 mm) were not different (*t* = 0.28, *df* = 77.5, *p* = 0.778) from those in Experiment 2 (18.1 ± 0.3 mm, 16–23 mm). There were no significant differences in the two behavioural traits (shyness: *t* = 1.30, *df* = 77.9, *p* = 0.196; activity: *t* = 1.66, *df* = 77.9, *p* = 0.100) between the two groups of males. The repeatability of shyness was 0.243 (95% credible interval: 0.005–0.438) for the males in Experiment 1 and 0.310 (95% credible interval: 0.069–0.559) for the males in Experiment 2. The repeatability of activity was 0.373 (95% credible interval: 0.092–0.607) for the males in Experiment 1 and 0.473 (95% credible interval: 0.225–0.691) for the males in Experiment 2. Shyness and activity were not significantly correlated at the within-individual level but were negatively correlated at both phenotypic and among-individual levels, with the latter indicating a strong shyness-activity behavioural syndrome (Table 2).

**Table 2:** The partitions of raw phenotypic correlations between shyness and activity of male mosquitofish *Gambusia affinis* used in Experiment 1 and 2, respectively

	Phenotypic correlation	Among-individual correlation	Within-individual correlation
Experiment 1	** − 0.453 (− 0.597, − 0.216)**	** − 0.958 (− 0.994, − 0.363)**	− 0.253 (− 0.498, 0.040)
Experiment 2	** − 0.241 (− 0.461, − 0.009)**	** − 0.937 (− 0.990, − 0.440)**	0.132 (− 0.201, 0.385)

Significant correlations were determined by the 95% credible intervals not including zero and are displayed in bold

*Phenotypic correlation* the correlation between individual mean values of two behavioural traits; among-individual correlation (= the repeatable component of phenotypic correlation, i.e., a behavioural syndrome): the individual average phenotypic responses of two traits are correlated; within-individual correlation: two traits show correlated changes within individuals.

### Mate choices

*Experiment 1* Although the neutral zone was larger than the preference zone in all the experiments, male mosquitofish spent more time in the preference zones. No overall directional preference of males for large (or small) females, with the same activity levels (active or inactive), was detected (Trial 1–1: 250.3 ± 18.8 s for large active females and 238.1 ± 18.1 s for small active females, *z* = -0.54, *df* = 78, *p* = 1; Trial 1–4: 231.0 ± 19.9 s for large inactive females and 217.2 ± 15.8 s for small inactive females, *z* =  − 0.62, *df* = 78, *p* = 1). Males spent more time associating with active females (Trial 1–2: 294.5 ± 24.7 s; Trial 1–3: 283.1 ± 17.1 s) than inactive females (Trial 1–2: 214.0 ± 23.3 s, *z* =  − 2.77, *df* = 78, *p* = 0.017; Trial 1–3: 201.2 ± 14.9 s, *z* = 4.13, *df* = 78, *p* < 0.001), regardless of whether they were large or small.

*Experiment 2* Consistent with Experiment 1, males in Experiment 2 spent more time in the preference zones. Males spent more time associating with active females (284.7 ± 21.1 s) than inactive females (206.0 ± 19.5 s; *z* = 3.24, *df* = 78, *p* = 0.004). Males increased their association time with inactive females (in Trial 2–1) when they were released to swim freely in Trial 2–2 (*t* = 2.03, *df* = 39, *p* = 0.049). This increase resulted in no difference in association time with the two females in Trial 2–2 (constantly active female: 231.2 ± 21.7 s; the female changing from inactive to active status: 246.9 ± 21.0 s; *z* =  − 0.60, *df* = 78, *p* = 1).

### Factors influencing male strength of preference

**Fig. 2:**
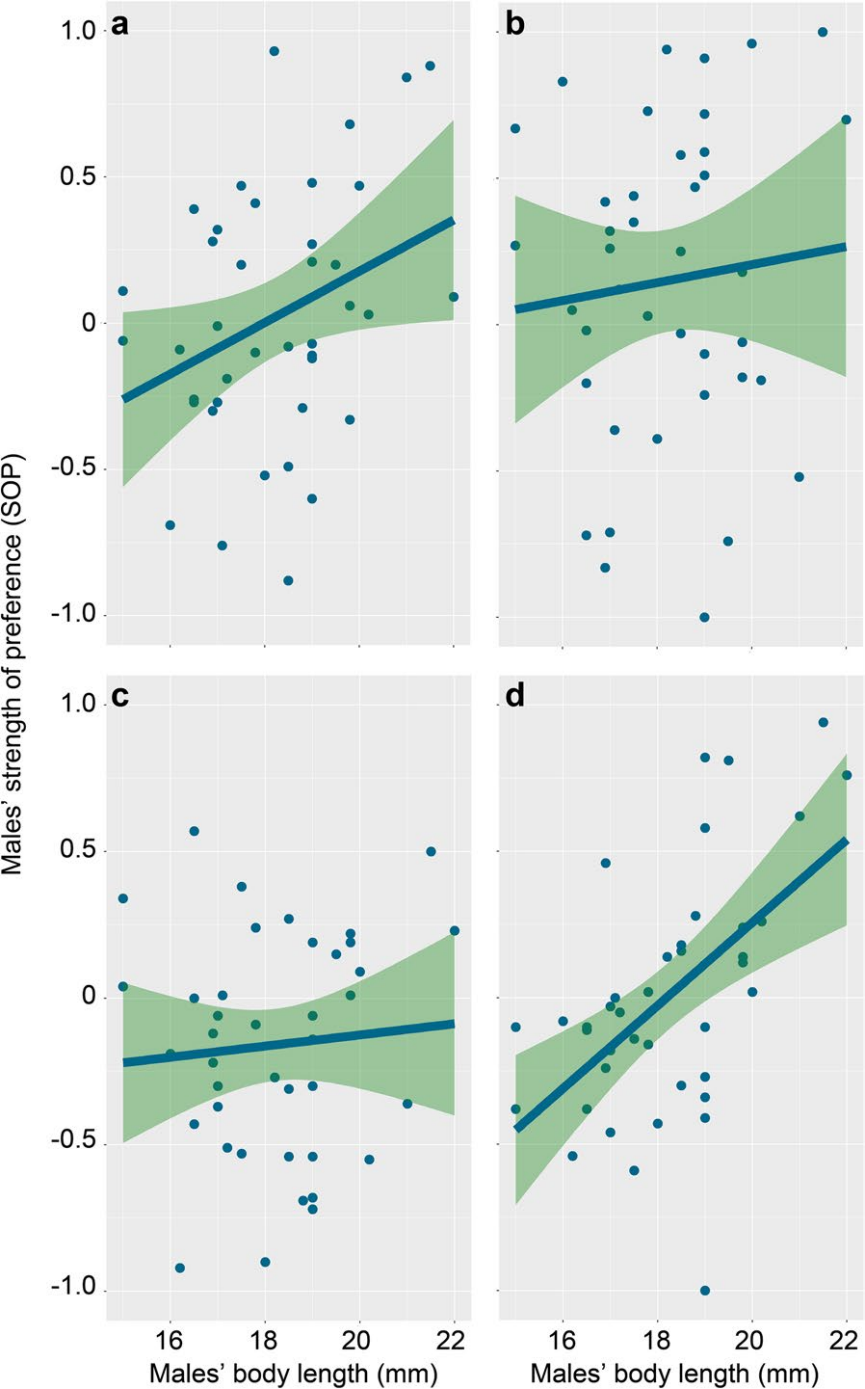
Strength of preference (SOP) of male mosquitofish *Gambusia affinis* for larger females as a function of males’ body length: **a** 22-mm active versus 18-mm active female (Trial 1–1), **b** 22-mm active versus 18-mm inactive female (Trial 1–2), **c** 22-mm inactive versus 18-mm active female (Trial 1–3), **d** 22-mm inactive versus 18-mm inactive female (Trial 1–4)

Males’ strength of preference (SOP) for larger females was positively correlated with males’ body length (Fig. 2) and the activity level of the larger females, but negatively with the activity level of the smaller females. When choosing from the paired females with the same body length, males’ SOP for active females was not significantly influenced by males’ personality traits and body length (Table 3).

**Table 3:** Effects of body length and personality traits (shyness and activity) of the male mosquitofish *Gambusia affinis* on the strength of their mating preference for larger females (Experiment 1; *R*^2^ = 0.156) and for active females (Trial 2–1; *R*^2^ = 0.161)

Experiment	Variables	Estimate	95% Credible intervals	*t* Value	*p* Value
Experiment 1	Male ID	0.0199	0.0000, 0.2507		
	Pair ID of stimulus females	0.0114	0.0000, 0.2418		
	Trial ID	0.0000	0.0000, 0.0875		
	Male’s shyness	− 0.0018	− 0.0040, 0.0004	− 1.663	0.096
	Male’s activity	− 0.0001	− 0.0004, 0.0001	− 1.061	0.289
	Male’s body length	0.0719	0.0186, 0.1256	2.745	**0.006**
	Activity of larger female (inactive)	− 0.1631	− 0.2845, − 0.0417	− 2.679	**0.007**
	Activity of smaller female (inactive)	0.1464	0.0250, 0.2678	2.404	**0.016**
Trial 2–1	Pair ID of stimulus females	0.0104	0.0000, 0.3509		
	Male’s shyness	− 0.0015	− 0.0051, 0.0021	− 0.850	0.395
	Male’s activity	0.0004	− 0.0001, 0.0008	1.700	0.089
	Male’s body length	0.0677	− 0.0171, 0.1541	1.655	0.098

Significant effects (*p* < 0.05) are displayed in bold

## Discussion

We tested whether mate choice exist in male mosquitofish and explored whether personality traits and body size of both sexes affect their mate preference*.* We found that male mosquitofish were highly selective when choosing a mate, providing further evidence for male mate choice being widespread in animals [9, 17]. In the wild, males often encounter more than one receptive female but are unable to mate with all of them at the same time. Differences in female quality and the gap between the number of available mates and the mating capacity of a male are often suggested as the factors governing male mate choice [6, 7]. As with females, an interesting question arises with male mate choice: are there certain phenotypic female traits that are driven evolutionarily by male mate choice? Compared with female mate choice, the evolutionary mechanism underlying male mate choice remains poorly understood despite some studies [29, 59]. In this study, we found that male mosquitofish choose mating partners according to females’ and activity level and body size and that females’ activity level may outweigh body size preference in male mate choice.

Body size is commonly highlighted in studies of mate choice because it is often positively linked with female fecundity and, thus, males choosing larger females are predicted to obtain higher reproductive fitness [17, 23]. Despite some studies reporting no preference for female body size [29], males in some live-bearing fish species have been found to preferentially mate with larger females [17, 19, 24], which was also expected in the present study. We found no overall directional preference of males for large (or small) females with the same activity levels. This finding is consistent with that of McPeek [60], who also found no overall preference for larger females. The overall non-directional pattern of preference for female body size was attributed to the fact that large females were preferred by large males while small females were selected by small males. Positive assortative mating by body size has been found in many species [22, 25, 61], and also in mosquitofish [60]. In poeciliid fish species characterized by promiscuity, males often sneakily follow females and attempt coerced copulations, while females often resist these males [43]. Larger males can better overcome the greater resistance of larger females to obtain the associated higher reproductive success. In the wild, larger males usually dominate the social hierarchies in populations and have more competitive advantages during mating [62, 63]. However, to have some chance of mating, smaller males must approach smaller females with weaker resistance ability [22].

We found convincing evidence that male mosquitofish preferred females with higher activity levels, regardless of their body size. Furthermore, when the enclosed female was released to swim actively, males increased their association time with it. Like body size, activity level has also been suggested to be linked with the fecundity potential of females because (1) activity is generally linked to metabolic processes, and thus higher activity level partly reflects better body condition [35, 64]; and (2) active females are more competitive than inactive individuals in foraging and intraspecific interactions, and thus can allocate more energy to their offspring [65]. Therefore, female activity level could be a criterion for males to use in choosing mating partners as males can increase their reproductive success by choosing active females. Furthermore, due to the benefits of higher activity level in females, males’ preference for larger females was found to be higher for females that were both larger and more active.

Consistent with Xu et al. [45], we found the existence of personality and behavioural syndrome in male mosquitofish from the same population. Some studies have found negative assortative preference related to behavioural traits and speculated that dissimilarity may increase behavioural compatibility between paired mating partners [66], and thus facilitate parental labour division, which may benefit offspring in biparental species [67]. Conversely, it has also been argued that cooperation between mating partners during the reproduction period could be promoted by behavioural synchronization (i.e., positive assortment), thus increasing reproductive success [61, 68]. In this study, we found that female activity might be a criterion for male mosquitofish to use while choosing mates. However, we did not find convincing evidence that males’ personality traits or body length affect their own preference for females’ activity level. To further explore this possible effect, a larger sample size might be needed.

## Conclusions

Our study suggests that both body size and personality traits play important roles in mate choice of male mosquitofish. There was no overall directional preference for large (or small) females with the same activity levels because larger males preferred larger females and smaller males chose smaller females. Males chose more active females, regardless of females’ body size and males’ preference for larger females was increased if the female was also more active. We did not find convincing evidence for the effect of males’ personality traits or body length on their preferences for females’ activity level. Body size is commonly highlighted in studies of mate choice; however, our study implies that personality traits may outweigh body size preferences in male mate choice.

## Supplementary Information

**Additional file 1.** Raw data of this study.

**Additional file 2.** R code for this study.

## Data Availability

The datasets supporting the conclusions of this article are included within the article and its Additional files 1, 2.
