# Supplementary material for: Male mate choice in mosquitofish: personality outweighs body size
Source: Front Zool. 2022 Jan 21;19:5. doi: 10.1186/s12983-022-00450-3 (PMC8780319; doi:10.1186/s12983-022-00450-3)
Supplement: Supplementary file 2 — Additional file 2. R code for this study. [file 12983_2022_450_MOESM2_ESM.docx]

**#The R codes used in the study “****Male mate choice in mosquitofish: personality outweighs body size”**

*# repeated-measures ANOVA to compare the association time in Expt 1*

library(reshape)

library(nlme)

exp1<-read.table("exp1.csv",header=T,sep=",")

shapiro.test(exp1$SOP1)

shapiro.test(exp1$SOP2)

shapiro.test(exp1$SOP3)

shapiro.test(exp1$SOP4)

trial1<-exp1[,1:4]

trial2<-exp1[,c(1,6:8)]

trial3<-exp1[,c(1,10:12)]

trial4<-exp1[,c(1,14:16)]

trial1<-melt(trial1, id="maleID")

trial2<-melt(trial2, id="maleID")

trial3<-melt(trial3, id="maleID")

trial4<-melt(trial4, id="maleID")

trial1$variable<-factor(trial1$variable, levels=unique(trial1$variable))

trial1$maleID<-factor(trial1$maleID, levels=unique(trial1$maleID))

trial2$variable<-factor(trial2$variable, levels=unique(trial2$variable))

trial2$maleID<-factor(trial2$maleID, levels=unique(trial2$maleID))

trial3$variable<-factor(trial3$variable, levels=unique(trial3$variable))

trial3$maleID<-factor(trial3$maleID, levels=unique(trial3$maleID))

trial4$variable<-factor(trial4$variable, levels=unique(trial4$variable))

trial4$maleID<-factor(trial4$maleID, levels=unique(trial4$maleID))

pre_trial1<-aov(value~variable+Error(maleID/variable), data=trial1)

pre_trial2<-aov(value~variable+Error(maleID/variable), data=trial2)

pre_trial3<-aov(value~variable+Error(maleID/variable), data=trial3)

pre_trial4<-aov(value~variable+Error(maleID/variable), data=trial4)

summary(pre_trial1)

summary(pre_trial2)

summary(pre_trial3)

summary(pre_trial4)

lme_trial1= lme(value ~ variable, data=trial1, random = ~1|maleID)

anova(lme_trial1)

summary(glht(lme_trial1,linfct=mcp(variable="Tukey")), test=adjusted(type="bonferroni"))

lme_trial2= lme(value ~ variable, data=trial2, random = ~1|maleID)

anova(lme_trial2)

summary(glht(lme_trial2,linfct=mcp(variable="Tukey")), test=adjusted(type="bonferroni"))

lme_trial3= lme(value ~ variable, data=trial3, random = ~1|maleID)

anova(lme_trial3)

summary(glht(lme_trial3,linfct=mcp(variable="Tukey")), test=adjusted(type="bonferroni"))

lme_trial4= lme(value ~ variable, data=trial4, random = ~1|maleID)

anova(lme_trial4)

summary(glht(lme_trial4,linfct=mcp(variable="Tukey")), test=adjusted(type="bonferroni"))

*# repeated-measures ANOVA to compare the association time in Expt 2*

library(reshape)

library(nlme)

exp2_1<-read.table("exp2_1.csv",header=T,sep=",")

exp2_2<-read.table("exp2_2.csv",header=T,sep=",")

exp21<-melt(exp2_1, id="maleID")

exp22<-melt(exp2_2, id="maleID")

exp21$variable<-factor(exp21$variable, levels=unique(exp21$variable))

exp22$variable<-factor(exp22$variable, levels=unique(exp22$variable))

pre_exp21<-aov(value~variable+Error(maleID/variable), data=exp21)

pre_exp22<-aov(value~variable+Error(maleID/variable), data=exp22)

summary(pre_exp21)

summary(pre_exp22)

lme_exp21= lme(value ~ variable, data=exp21, random = ~1|maleID)

anova(lme_exp21)

summary(glht(lme_exp21,linfct=mcp(variable="Tukey")), test=adjusted(type="bonferroni"))

lme_exp22= lme(value ~ variable, data=exp22, random = ~1|maleID)

anova(lme_exp22)

summary(glht(lme_exp22,linfct=mcp(variable="Tukey")), test=adjusted(type="bonferroni"))

# paired t-test to investigate the change in males’ association time with the inactive female in Trial 2-1 after it was released to be active in Trial 2-2

exp2_1to2<-read.table("exp2_1to2.csv",header=T,sep=",")

t.test(exp2_1to2$SOP1, exp2_1to2$SOP2, paired=T)

*#calculation of behavioral repeatability and correlation*

library("MCMCglmm")

library(ggplot2)

pers1R<-read.table("pers1R.csv",header=T,sep=",")

pers2R<-read.table("pers2R.csv",header=T,sep=",")

pers1R$shy<-log(pers1R$shy)

pers1R$expl<-log(pers1R$expl)

pers2R$shy<-log(pers2R$shy)

pers2R$expl<-log(pers2R$expl)

prior = list(R = list(V = diag(2), n = 0.01),
             G = list(G1 = list(V = diag(2), n = 0.01)))

corr1<- MCMCglmm(cbind(shy,  expl) ~ trait-1, random = ~ us(trait):maleID, rcov = ~us(trait):units,  prior=prior, family = rep("gaussian", 2), nitt = 220000, burnin = 20000, thin=25, data = pers1R, verbose = FALSE)

corr2<- MCMCglmm(cbind(shy,  expl) ~ trait-1, random = ~ us(trait):maleID, rcov = ~us(trait):units,  prior=prior, family = rep("gaussian", 2), nitt = 220000, burnin = 20000, thin=25, data = pers2R, verbose = FALSE)

colnames(corr1$VCV)

posterior.mode(posterior.cor(corr1$VCV[,1:4]))

HPDinterval(posterior.cor(corr1$VCV[,1:4]))

posterior.mode(posterior.cor(corr1$VCV[,5:8]))

HPDinterval(posterior.cor(corr1$VCV[,5:8]))

pheno.cor1 <-as.mcmc(cbind(rowSums(corr1$VCV[,c(1,5)]), rowSums(corr1$VCV[,c(2,6)]) , rowSums(corr1$VCV[,c(3,7)]) , rowSums(corr1$VCV[,c(4,8)])))

posterior.mode(posterior.cor(pheno.cor1))

HPDinterval(posterior.cor(pheno.cor1))

posterior.mode(posterior.cor(corr2$VCV[,1:4]))

HPDinterval(posterior.cor(corr2$VCV[,1:4]))

posterior.mode(posterior.cor(corr2$VCV[,5:8]))

HPDinterval(posterior.cor(corr2$VCV[,5:8]))

pheno.cor2 <-as.mcmc(cbind(rowSums(corr2$VCV[,c(1,5)]), rowSums(corr2$VCV[,c(2,6)]) , rowSums(corr2$VCV[,c(3,7)]) , rowSums(corr2$VCV[,c(4,8)])))

posterior.mode(posterior.cor(pheno.cor2))

HPDinterval(posterior.cor(pheno.cor2))

rep1<-as.mcmc(cbind(corr1$VCV[,1]/(corr1$VCV[,1]+corr1$VCV[,5]), corr1$VCV[,4]/(corr1$VCV[,4]+corr1$VCV[,8])))

posterior.mode(rep1)

HPDinterval(rep1)

rep2<-as.mcmc(cbind(corr2$VCV[,1]/(corr2$VCV[,1]+corr2$VCV[,5]), corr2$VCV[,4]/(corr2$VCV[,4]+corr2$VCV[,8])))

posterior.mode(rep2)

HPDinterval(rep2)

*#generalized linear mixed-effects models to test the effects of factors on males’ mate preference*

library(lme4)

library(car)

library(arm)

exp1<-read.table("exp1.csv",header=T,sep=",")

shapiro.test(exp1$SOP)

exp11<-exp1[which(exp1$trialID==1),]

exp2<-read.table("exp2.csv",header=T,sep=",")

shapiro.test(exp2$SOP)

exp21<-exp2[which(exp2$trialID==1),]

t.test(exp11$length, exp21$length)

t.test(exp11$shy, exp21$shy)

t.test(exp11$expl, exp21$expl)

lm1<-lmer(SOP~ shy+expl+length+f1+f2 +(1|femaleID) +(1|trialID) +(1|maleID), data=exp1, REML = FALSE)

summary(lm1)

Anova(lm1)

lm21<-lmer(SOP~ shy+expl+length+ (1|femaleID), data= exp21, REML = FALSE)

summary(lm21)

Anova(lm21)

confint.merMod(lm1)

confint.merMod(lm21)
